# Development and Evaluation of the Virtual Pathology Slide: A New Tool in Telepathology

**DOI:** 10.2196/jmir.5.2.e11

**Published:** 2003-06-13

**Authors:** Sean SP Costello, Daniel J Johnston, Peter A Dervan, Daniel G O'Shea

**Affiliations:** ^1^School of BiotechnologyMedical Informatics GroupDublin City UniversityDublinIreland; ^2^Mater Misericordiae HospitalThe Conway Institute of Biomolecular and Biomedical ResearchUniversity College DublinThe Pathology DepartmentDublinIreland

**Keywords:** Telepathology, Internet, telemicroscopy, remote diagnosis, virtual slide, pathology, imaging

## Abstract

**Background:**

The Virtual Pathology Slide is an interactive microscope emulator that presents, via the Internet or CD-ROM, a complete 15.53 mm x 11.61 mm digitalized tissue section. The Virtual Pathology Slide mimics the use of a microscope in both the stepwise increase in magnification (from 16x up to 2000x) and in lateral motion in the X and Y Cartesian directions. This permits a pathologist to navigate to any area on a slide, at any magnification, similar to a conventional microscope.

**Objective:**

The aim of this study was to assess the diagnostic accuracy and acceptability of the Virtual Pathology Slide.

**Methods:**

Ten breast needle core biopsies were randomly selected and presented to 17 pathologists or trainee pathologists with at least 2 years experience in pathology practice. Participants were required to examine each case online and provide a diagnostic classification using online feedback forms. The recorded data permitted examination of interobserver variability and user satisfaction.

**Results:**

Agreement between original glass-slide diagnosis and consensus diagnosis using the Virtual Pathology Slide was reached in 9 out of 10 slides. Percentage concordance for slides lay in the range of 35.3% to 100% with an average percentage concordance between slides of 66.5%. The average Kappa statistics for interobserver agreement was 0.75 while average percentage concordance amongst participants was 66.5%. Participants looked at an average of 22 fields of view while examining each slide. Confidence: 81.25% of the participants indicated confidence using the Virtual Pathology Slide to make a diagnostic decision, with 56.25% describing themselves as "reasonably confident," 18.75% as "confident," and 6.25% as "very confident." Ease of use: 68.75% reported the system as "easy" or "very easy" to use. Satisfaction: 87.5% of participants expressed satisfaction with image quality, with 43.75% describing the image quality as "adequate," 25% describing it as "good," and 18.75% describing the image quality as "excellent." Pathologists with a working bandwidth greater than 20 kilobits per second found the download speed of the Virtual Pathology Slide "adequate" or better.

**Conclusions:**

Results from this study show that the Virtual Pathology Slide can be used to make a correct diagnostic decision, and that the system is a realistic alternative to dynamic telepathology.

## Introduction

### Definition of Telepathology

Telepathology is the practice of diagnostic pathology by a remote pathologist utilizing images of tissue specimens transmitted over a telecommunications network [1,2]. Traditionally telepathology systems are defined as either dynamic or static. Dynamic systems allow a telepathologist to view images transmitted in real time from a remote robotic microscope that permits complete control of the field of view and magnification [3]. Static (or store-and-forward) telepathology involves the capture and storage of images followed by transmission over the Internet via e-mail attachment, file transfer protocol, or a Web page, or distribution via CD-ROM. Dynamic hybrids also exist, which incorporate aspects of both technologies [3].

### Applications of Telepathology

The diversity in telepathology systems reflects growing technological expertise in this area and the increasing importance of telepathology in education, training, quality assurance, and teleconsultation [4- 9]. Numerous pathology archives abound on the Internet providing links to both educational and commercial telepathology websites. These offer access to either static or dynamic image delivery systems [7- 19].

### Limitations of Telepathology

Image quality and the ability to make diagnostic decisions from electronically-compressed images is a contentious issue [3,19- 20]. In order for telepathology to be of clinical use, studies have attempted to access the diagnostic accuracy of store-and-forward telepathology, and have shown accuracy in the range of 77% to 100% [3,20- 28]. The diverse nature of this technology makes it difficult to draw comparisons between studies, or to form a consensus on a method of best practice. There is no universally-accepted standardization in hardware, software, image resolution, color-depth, or image compression and storage [3]. However, studies have shown that the use of images with as low a resolution as 1024 pixels x 768 pixels resolution x 24-bit color does not impair diagnostic performance [3,20,27- 29]. To contend with such nonstandardization, guidelines have been formulated for the capture and treatment of diagnostic images and for the practice of telepathology [30- 31].

Recent improvements in Internet-browser technology have facilitated the development of interactive store-and-forward Web pages. These feature the ability to show the spatial relationship between individual images in low-power and high-power views. This technology is commonly visualized using a small image gallery constructed from one or two microscopic fields out of a possibility of thousands, displaying images of the same fields at higher magnifications [3,20,21]. Field selection and interpretation are thought to be the primary reasons specific to store-and-forward telepathology that account for its discordance with diagnosis in a conventional pathology setting [19- 20]. Studies involving multiple pathologists provide the most robust and accurate method of assessing a telepathology technique [20- 27]. However it is difficult to distinguish the performance of the technology from the skill of the pathologist and the degree of difficulty of the cases being presented [21]. Until recently, the development of a tool for routine diagnosis and teleconsultation was the driving goal for the evolution of telepathology systems. Initial expense, lack of broadband Internet connections, potential liabilities, and a lack of knowledge transfer from expert to potential user have all contributed to preventing the incorporation of telepathology into everyday practice [32- 35]. The emerging role of telepathology in the area of education and quality assurance is not encumbered by the same difficulties. It has been demonstrated that the application of telepathology in such roles has the advantage of lower cost, less logistical effort, and a positive response to its use by the end user [36- 39]. Coupled with the growing presence of ultra-fast slide scanners, this should ensure an increasing role for telepathology in this area [40- 42].

### The Virtual Pathology Slide (VPS)

**Figure 1:**
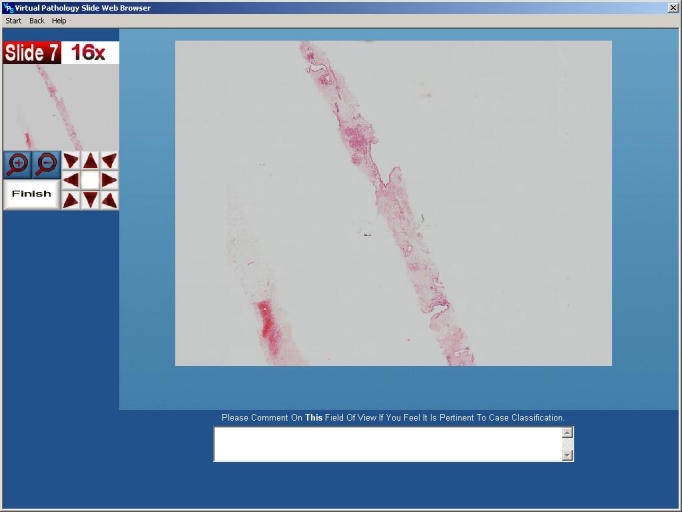
Screenshot of the Virtual Pathology Slide Web browser (for further screenshots, see Multimedia Appendix 1)

To overcome problems attributable to sampling bias and interpretation resulting from limited field selection, telepathologists must be able to navigate to any field of view, at magnifications comparable to that of a conventional microscope, using images of sufficient resolution to render a correct diagnosis [21,22,35]. To meet such criteria we have developed the Virtual Pathology slide (VPS) [43,44]. This is a microscope emulator that displays digitized representations of tissue slides, allowing inspection of numerous fields of view, over a wide range of magnifications. Similar applications, commonly referred to as Virtual Slides, have been developed by other commercial and academic bodies [16,18,20,34,35,40- 42]. A screenshot of our VPS is shown in Figure 1; further screenshots are in Multimedia Appendix 1.

In an important new departure, the VPS can also record and quantify the diagnostic trace of a pathologist, as a discrete data set on a central server. This allows the decoupling of a pathologist's field selection from the technical functionality of the telepathology system. In this paper, we report on the development of this system, its acceptability among a group of evaluating pathologists, the level of diagnostic agreement among this group, and the potential future applications of the VPS in telepathology.

## Methods

A comprehensive document detailing the scanning algorithm and system architecture of the VPS is in Multimedia Appendix 2.

### Construction of the VPS

#### Development of VPS Imaging Workstation

To create VPS slides, an imaging workstation was developed in-house. An Olympus BX-40 microscope (Olympus, Melville, NY, USA) incorporating a 40x plan apochromat lens with a 0.95 numerical aperture was used. The microscope was fitted with a robotic stage (Prior Scientific Inc, Rockland, Mass, USA) and a JVC 3-CCD (3-chip charge-coupled device) video camera.

#### Development of VPS Slide Scanning Algorithm

Using Optimas 6.5 imaging software (Media Cybernetics, Inc, Silver Spring, Md, USA), an algorithm was written in ALI (Analytical Language for Images) to perform a raster scan of 15.53 mm x 11.61 mm (180 mm ^2^) of tissue at 40x objective magnification. The VPS raster scan acquires 128 x 128 images in the X and Y Cartesian directions, one row at a time. Each acquired image represents 0.011 mm ^2^ at a resolution of 768 pixels by 574 pixels. Images were saved using a JPEG (Joint Photographic Experts Group) format at 10% compression, resulting in image-file sizes in the range of 100 to 150 KB (kilobytes). To build layers of lesser magnification, a second algorithm was developed, which tiles and resizes multiple images from the raster scan into composite images [16]. Images were subsequently uploaded onto the VPS Web server.

#### Development of VPS Web Interface

To view images via the Internet a graphical user interface was constructed [45]. This is a Web page powered by server-side scripting in PHP (PHP = Hypertext Preprocessor). The interface emulates the experience of using a conventional microscope by allowing a user to increase or decrease magnification or move laterally while examining a tissue section.

A customized browser was developed to control the user's access to the VPS during dedicated studies, to optimize the integrity of recorded data, and to provide a uniform experience for users who would otherwise experience subtle differences due to variation in currently-existing versions of Web browsers.

The VPS browser is a Microsoft Foundation Class (MFC) application written in Visual C++, which utilizes Internet Explorer file libraries to behave as a customized browser. The VPS customized browser opens up prescribed Web pages on the VPS server. The VPS browser is optimized for PC users with Microsoft Internet Explorer 5 or greater.

#### Development of VPS Database

When a user examines a VPS slide, data describing the user's interaction with the VPS is transmitted from the user's workstation to the VPS server and stored in an Oracle database. The VPS examination database is structured to contain the following data types:

#### System Configuration Data

This consists of data automatically recorded on the VPS server and includes parameters such as user's browser version, operating system, screen resolution, screen color depth, and IP (Internet Protocol) address.

#### User Tracking Data

This data records a user's "diagnostic pattern" as the user examines a slide. Information recorded includes image file name, image magnification, and the time spent viewing each image.

#### User-submitted Data

Diagnostic and descriptive data is submitted to the VPS server by participants, using HTML (Hypertext Markup Language) forms. Information recorded includes the report submitted by the user at the end of each slide examination and a final questionnaire. The observer also has the option to record or annotate every field of view examined.

#### VPS Deployment

The user has two choices on how he or she wishes to use the system. Users with high-speed Internet access can download the VPS browser from the VPS homepage and view images downloaded directly from the VPS server. To accommodate users with slow Internet connections, users may launch the VPS browser from a VPS CD and view images stored on the CD. However, an Internet connection is still required to record data on the VPS database, and to provide essential data for statistical analysis and playback facilities.

### Validation of the VPS

#### Slide Selection

**Figure 2:**
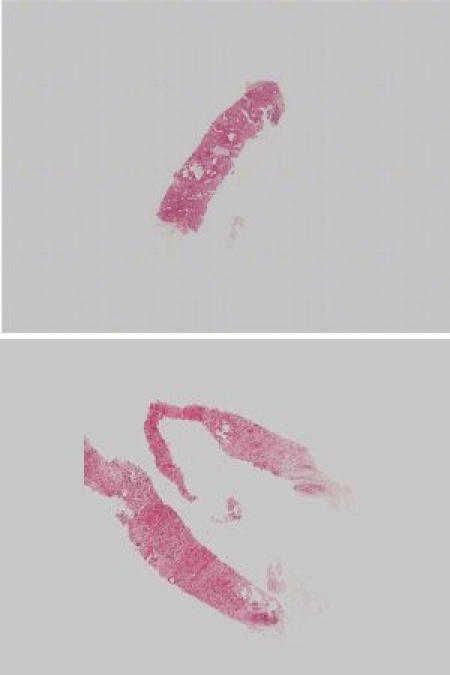
Two examples of the 10 breast needle core biopsies presented to 17 pathologists or trainee pathologists using the VPS (for all 10 images, see Multimedia Appendix 3)

Ten needle core biopsies were obtained from the Department of Pathology, Mater Misericordiae Hospital, Dublin, Ireland. The slides were randomly selected by a pathologist (P.A.D.) with a special interest in breast pathology. The slides represented a range of diagnostic classifications. Two of the slides are presented in Figure 2. All 10 slides can be viewed in Multimedia Appendix 3.

#### Participants

Fifty-four pathologists with at least 2 years experience in pathology practice registered for the study. Of the 54 pathologists, 17 examined all 10 slides and 8 initiated the study but did not complete it. Of the 17 participants who completed the study, 8 were members of the European Working Group of Breast Screening Pathology. Of the 17 participants who examined all 10 slides, 13 subsequently completed a questionnaire on user perception of the VPS. Of the 8 participants that initiated the study but did not complete it, 3 completed the questionnaire.

#### Examination Procedure

Upon launching the VPS browser, participants were prompted to log in using the username and password they received at registration. This made them identifiable to the system. On successful log-in, the VPS needle core examination guidelines [46] were displayed.

After stating they read the guidelines, users were permitted to browse the slides available for examination and select one from a slide gallery. The slide gallery displayed a thumbnail image of each slide and indicated the patient's age and sex, and a brief case description.

Upon selecting a slide for examination, participants were presented with the VPS user interface. While examining a slide, participants could if desired annotate the fields of view using the text area provided. Upon completing a slide examination, participants submitted an online report that provided a diagnostic classification for the case, using an adaptation of the Core Biopsy Reporting Guidelines for Non-operative Diagnostic Procedures and Reporting in Breast Cancer screening [47] as used by the British National Co-ordinating Committee for Breast Screening Pathology. Users were requested to classify the slides as one of the following:

B1: Unsatisfactory/normal tissue only.

B2: Benign.

B3: Benign but of uncertain malignant potential.

B4: Suspicious of malignancy.

B5: Malignant.

For slides categorized as B5, participants were required to subclassify their decision as malignant, in-situ, or invasive. Upon making a classification, participants were returned to the slide gallery from which another slide could be selected for examination.

Utilization of this data allowed the following to be determined:

- Percentage concordance for a user, calculated as the number of slides (expressed as a percentage) for which the user's diagnosis is in agreement with the consensus VPS diagnosis.
- Percentage concordance of a slide, calculated as the percentage of users who concur as to the correct diagnosis of a slide.
- Cohen's Kappa [48- 49], a measure of agreement between observers taking into account agreement that could occur by chance. Kappa values range from 0 to 1 with a score greater than 0.7 indicating "substantial agreement."

Participants who completed examination of the 10 slides were subsequently requested to complete an online questionnaire describing their experience using the VPS. Participants were asked to give a subjective evaluation of diagnostic confidence in using the VPS, reasons for uncertainty, an evaluation of image quality, and perceived download speed.

## Results

### User Performance Using the VPS

Table 1 shows strong diagnostic agreement between original glass-slide diagnosis and the most-common diagnosis offered by users of the VPS, with agreement being reached in 9 out of the 10 slides. Disagreement by 1 diagnostic degree occurred with slide 8 (glass slide diagnosis was B3; most- common VPS diagnosis was B4). The diagnostic classification of slide 8 had the lowest level of agreement between participants at 38.5%. The second most popular choice for slide 8 was split between B3 and B2, 6 participants (35.3% of users) classified it as B4 while 4 participants (23.5% of users) classified it as B3 and 4 participants (23.5% of users) classified it as B2). Participants with the 4 highest Kappa scores (23.5% of users) classified slide 8 as B4.

**Table 1:** Comparison of glass slide needle core surgical biopsy diagnosis and most-common Virtual Pathology Slide (VPS) diagnosis, in order of level of agreement (concordance) for each slide

	**Virtual Pathology Slide**									
	**S6**	**S2**	**S3**	**S4**	**S7**	**S9**	**S10**	**S1**	**S5**	**S8**
Diagnosis Glass	B5	B5	B5	B2	B2	B2	B2	B5	B5	B3
Diagnosis VPS	B5	B5	B5	B2	B2	B2	B2	B5	B5	B4
Concordance, %	100	94.1	82.4	76.5	64.7	58.8	52.9	52.9	47.1	35.3
Fields of view*	243	208	464	410	463	309	591	431	487	299

A more-detailed analysis of the diagnostic classifications made by participants is described in Table 2. The average percentage concordance between participants on all cases was 66.5%. Of the 17 participants, 14 attained a percentage concordance of between 90% and 60%.

**Table 2:** For each participant: years of experience in pathology practice, diagnostic classification of slides, level of agreement with each other (% concordance and Kappa index), and number of fields of view examined

ID*	**EXP**†	**Virtual Pathology Slide**										**Concordance,%**‡	**Kappa**§	**Fields of View**
		**S6**	**S2**	**S3**	**S4**	**S7**	**S9**	**S10**	**S1**	**S5**	**S8**			
5	5	B5	B5	B5	B2	B2	B2	B2	B4	B5	B4	90	0.97	321
62	5	B5	B5	B5	B2	B2	B3	B2	B5	B4	B4	80	0.94	326
35	5	B5	B5	B5	B2	B2	B1	B1	B4	B5	B4	70	0.94	122
10	5	B5	B5	B5	B2	B3	B3	B2	B5	B4	B4	70	0.91	157
39	5	B5	B5	B5	B1	B3	B3	B2	B5	B5	B5	60	0.90	343
55	5	B5	B5	B5	B2	B2	B2	B2	B5	B3	B3	80	0.87	289
87	5	B5	B5	B5	B1	B2	B2	B2	B3	B5	B3	70	0.86	130
18	3	B5	B5	B4	B1	B3	B2	B1	B5	B4	B3	40	0.86	252
68	5	B5	B5	B5	B2	B3	B2	B2	B5	B4	B2	70	0.85	228
65	5	B5	B5	B5	B2	B2	B3	B1	B3	B4	B4	60	0.80	234
22	5	B5	B5	B5	B2	B2	B2	B5	B5	B5	B5	80	0.75	204
41	5	B5	B5	B5	B1	B2	B2	B2	B2	B4	B4	70	0.75	216
7	5	B5	B5	B5	B2	B2	B2	B1	B4	B2	B3	60	0.73	121
1	5	B5	B5	B4	B2	B4	B2	B2	B5	B5	B1	70	0.67	223
75	5	B5	B5	B5	B2	B2	B2	B5	B4	B5	B2	70	0.65	120
36	3	B5	B5	B2	B2	B3	B3	B4	B5	B2	B2	40	0.26	201
6	5	B5	B2	B5	B2	B2	B4	B5	B3	B5	B2	50	0.23	418
**Average**												66.5	0.76	23

^*^ ID = identification number of participant.

^†^ EXP = years of experience in pathology practice.

^‡^ Concordance = number of slides (expressed as a percentage) for which the user's diagnosis is in agreement with the consensus Virtual Pathology Slide diagnosis.

^§^ Kappa = Cohen's Kappa, a measure of agreement between observers, taking into account agreement that could occur by chance. Kappa greater than 0.7 indicates "substantial agreement."

The average Kappa value achieved by participants was 0.76. Participants 36 and 6 achieved a Kappa of 0.26 and 0.23 respectively indicating "fair agreement" [31- 32] with other participants while the remaining 15 participants achieved a Kappa of between 0.97 and 0.65.

The average percentage concordance for slides was 66.5% with a minimum concordance of 35.3% for slide 8 and a maximum concordance of 100% for slide 6. The percentage concordance for slide 5 was 47%. For all remaining slides there was greater than 50% agreement between participants.

The average number of fields of view examined by each participant was 23 per slide. Participant number 5, who achieved the highest Kappa, examined 321 views, while participant number 6, who had the lowest Kappa, examined 418 fields of view.

The highest number of number of fields of view examined for a particular slide was 118 by participant number 6 while examining slide 10. This slide had a percentage concordance between participants of 52.9%. The lowest number of views examined while examining a slide was 3; this was by participant 10 who achieved a Kappa score of 0.91 and agreed with the group consensus for slide 2. Diagnosis for slide 2 had a percentage concurrence amongst participants of 94%.

The average time taken for participants to examine a slide was recorded as 6 minutes 11 seconds. The maximum time taken to examine a slide was recorded as 12 minutes 49 seconds by participant number 36 with an average bandwidth of 20 kilobits per second while examining slide 7. The minimum examination time was recorded as 43 seconds by participant number 1 with an average bandwidth of 64 kilobits per second while examining slide 2.

### User Perception of the VPS

Participants were asked to assess their own computer competency and the frequency with which they use a telepathology system. Participants described themselves as "advanced" (18.75%), "competent" (18.75%), or "adequately competent" (62.5%) with computers, while 44% of participants indicated they had never used a telepathology system prior to the study.

**Figure 3:**
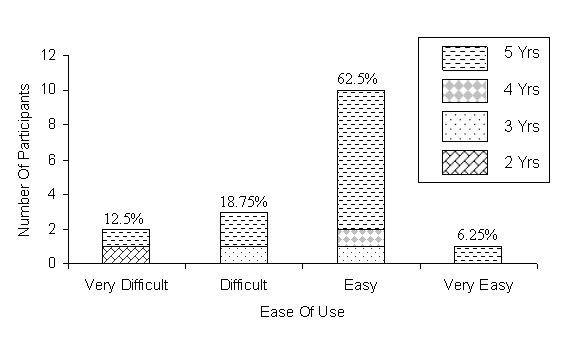
Ease of use of the Virtual Pathology Slide (VPS). Yrs = years of pathology experience. Percentages = percentage of 16 participants for that rating

**Figure 4:**
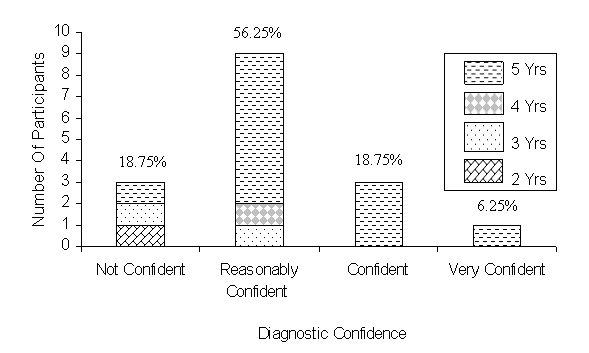
Degree of confidence in using the Virtual Pathology Slide (VPS) to make a diagnostic decision. Yrs = years of pathology experience. Percentages = percentage of 16 participants for that rating

Figure 3 illustrates that 68.75% of participants rated the VPS "easy" (62.5%) to use or "very easy" to use (6.25%). Participants were requested to rate their degree of confidence in making a diagnostic decision using the VPS.

Figure 4 illustrates that 80.25% of participants expressed confidence in using the VPS with 56.25% indicating they were "reasonably confident," while 18.75% were "confident," and 6.25% were "very confident" in making a diagnosis.

**Figure 5:**
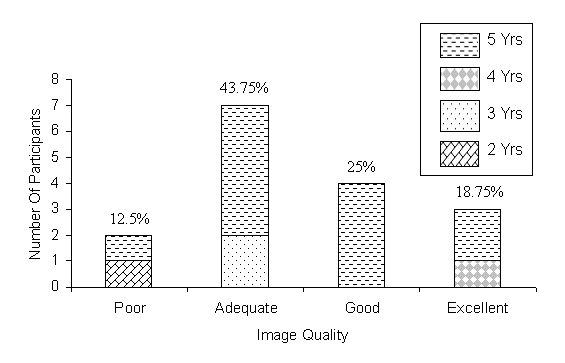
Perceived image quality of the Virtual Pathology Slide (VPS). Yrs = years of pathology experience. Percentages = percentage of 16 participants for that rating

Figure 5 illustrates that 87.5% of participants expressed satisfaction with the image quality with 43.75% indicating the quality as "adequate," 25% as "good," and 18.75% of participants indicating the image quality as "excellent."

## Discussion

The VPS system is a realistic alternative to dynamic telepathology, in terms of its ability to mimic a conventional microscope, its accessibility via the Internet, and its simplicity of operation. Of the 17 participants, 15 achieved a Kappa of between 0.97 and 0.65 and 14 attained a percentage concordance of between 90% and 60%. This demonstrates "substantial" agreement between users when using the VPS [31- 32]. The calculation of Kappa was weighted to reflect the degree of variation of a participant's diagnostic decision from the most popular choice. For example, participant 18 achieved a high Kappa of 0.86 despite being in agreement with other participants for 4 out of the 10 slides. This is because for each of the other 6 slides, participant 18 was inconsistent with the popular choice by one degree. Participant 36 achieved the same percentage concordance as participant 18 but only achieved a Kappa of 0.26. This is because the diagnostic categories selected by participant 36 deviated to a greater degree from the popular choice than those selected by participant 18 [48- 49].

Participant 36 and participant 6 attained the lowest Kappa scores of 0.26 and 0.23 respectively. This reduced the overall average Kappa value considerably. Confidence in using the VPS was described as "reasonably confident" by participant 36, who had 3 years experience in pathology and examined 201 fields of view while examining the entire set of slides. Further analysis of the images viewed is necessary to elucidate reasons for the diagnostic decisions made by participant 36; however, inexperience with breast pathology coupled with insufficient examination of the slides may have contributed to poor performance. Use of telpathology was described as "infrequently" by participant 6 who was "confident" in making a diagnostic classification using the VPS and described the use of the VPS as "easy." However, participant 6 attributed some diagnostic uncertainty to "problems with assessing significance of small subtle lesions without having the whole slide to look at." Participant 6 examined 418 fields of view, the highest number examined by any participant.

The average percentage concordance for the entire set of slides was 66.5%. Full agreement between participants was achieved for slide 6, which demonstrates that full agreement can be achieved using the VPS.

The average number of views examined by participants while examining the entire set of slides was 230. The percentage concordance for a particular slide decreases as the average number of fields examined for that slide increases. For example, the average number of fields examined for slide 6 (100% concordance amongst participants) was 14.3, while the average number of fields examined for slide 10 (52.9% concordance amongst participants) was 34.8. Conversely, participants with a high Kappa score tend to view a greater number of fields of view than participants with a low Kappa score, suggesting that the greater the amount of tissue viewed by a pathologist, the more likely they are to make a correct diagnosis.

Slide 8 had the lowest level of concordance at 35.3%. This reduced the average percentage concordance for the set of slides by 3.46%. Table 2 shows there is a broad distribution of diagnostic categorization for slide 8 by participants. As shown in Table 1, for slide 8 the number of fields of view examined by participants is low (299) given the apparent complexity of the case. It is apparent that users are rapidly coming to a conclusion that usually does not concur with the original glass slide diagnosis. Further study of the examination traces from this slide will be required to evaluate the reasons for the diagnostic spread.

Participants with 3 years or less experience did not have access to broadband Internet connection and recorded bandwidth speeds of less than 15 kilobits per second. These participants expressed least satisfaction with the VPS in terms of ease of use, image quality, and diagnostic confidence. All 3 participants, who indicated they were "not confident," attributed difficulty in using the VPS to poor download speed, with comments such as "Poor download speed was extremely slow and made the viewing experience disjointed and basically unworkable." Of these 3 participants, 2 had a working bandwidth of 12.6 kilobits per second and 31.5 kilobits per second respectively. A bandwidth could not be determined for the third, however the third did offer such comments as "too long to download images" and "problem was on my end, slow connection." High-speed broadband Internet connectivity is still unavailable to many pathologists. This is a major limiting factor for acceptability of Web-driven telepathology due to the time taken to download large image files over the Internet [2,4,39]. We have attempted to overcome this with the development and deployment of a CD-ROM VPS system to selected participants. This facilitates rapid retrieval of images from a CD while data pertaining to the examination is transmitted and stored on the VPS web server.

Participants were asked to comment on improvements to the VPS that they would like implemented. A number of participants suggested they would like additional magnification ranges. For example, "Navigation within the slide was disjointed and it was difficult to maintain perspective whilst moving from field to field. The range of magnifications was too limited, especially in the intermediate magnification range."

There are a growing number of interactive pathology sites available via the Internet [7- 19]. The diversity in their principle of operation, their application in telepathology, and their degree of sophistication promises an encouraging future in telepathology. The contribution of the VPS to the field of telepathology is notable in that it records the diagnostic pathway of a pathologists slide examination. We now have the diagnostic traces of 17 pathologists examining 10 cases. We intend to utilize this data to elucidate the cognitive and decision-making process of pathologists as they render a diagnosis when using a microscope. This will provide valuable insight into interobserver variability and the subjective process of microscopic diagnosis.

## Abbreviations

VPS: Virtual Pathology Slide

## Multimedia Appendix 1

Screenshots of the Virtual Pathology Web browser.

## Multimedia Appendix 2

Technical Documentation.

## Multimedia Appendix 3

Ten breast needle core biopsies presented to 17 pathologists or trainee pathologists using the VPS.
