# Supplementary material for: Development and Evaluation of the Virtual Pathology Slide: A New Tool in Telepathology
Source: J Med Internet Res. 2003 Jun 13;5(2):e11. doi: 10.2196/jmir.5.2.e11 (PMC1550558; doi:10.2196/jmir.5.2.e11)

## Slide 1
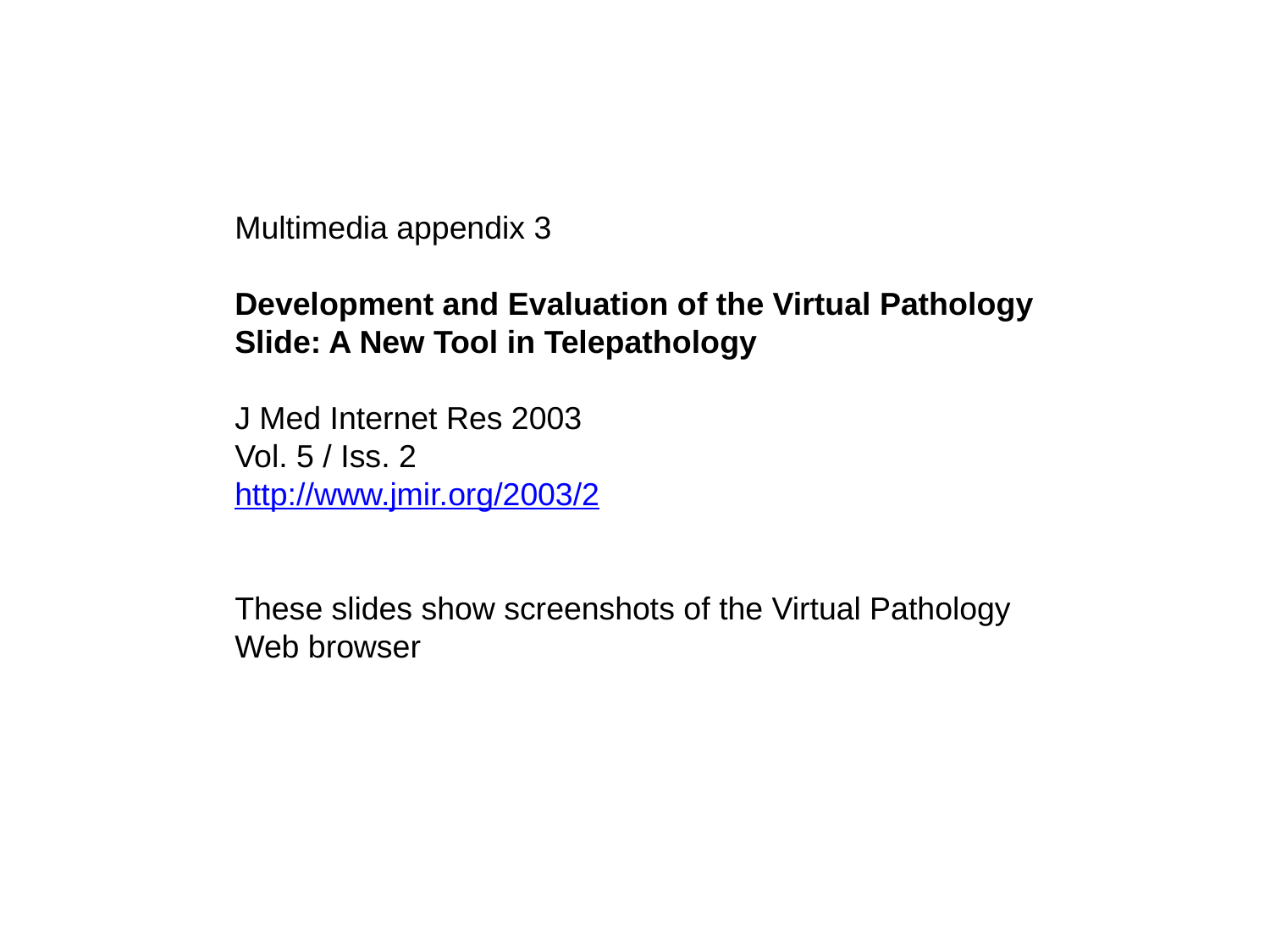

Multimedia appendix 3
Development and Evaluation of the Virtual Pathology Slide: A New Tool in Telepathology
J Med Internet Res 2003
Vol. 5 / Iss. 2
http://www.jmir.org/2003/2
These slides show screenshots of the Virtual Pathology Web browser

## Slide 2
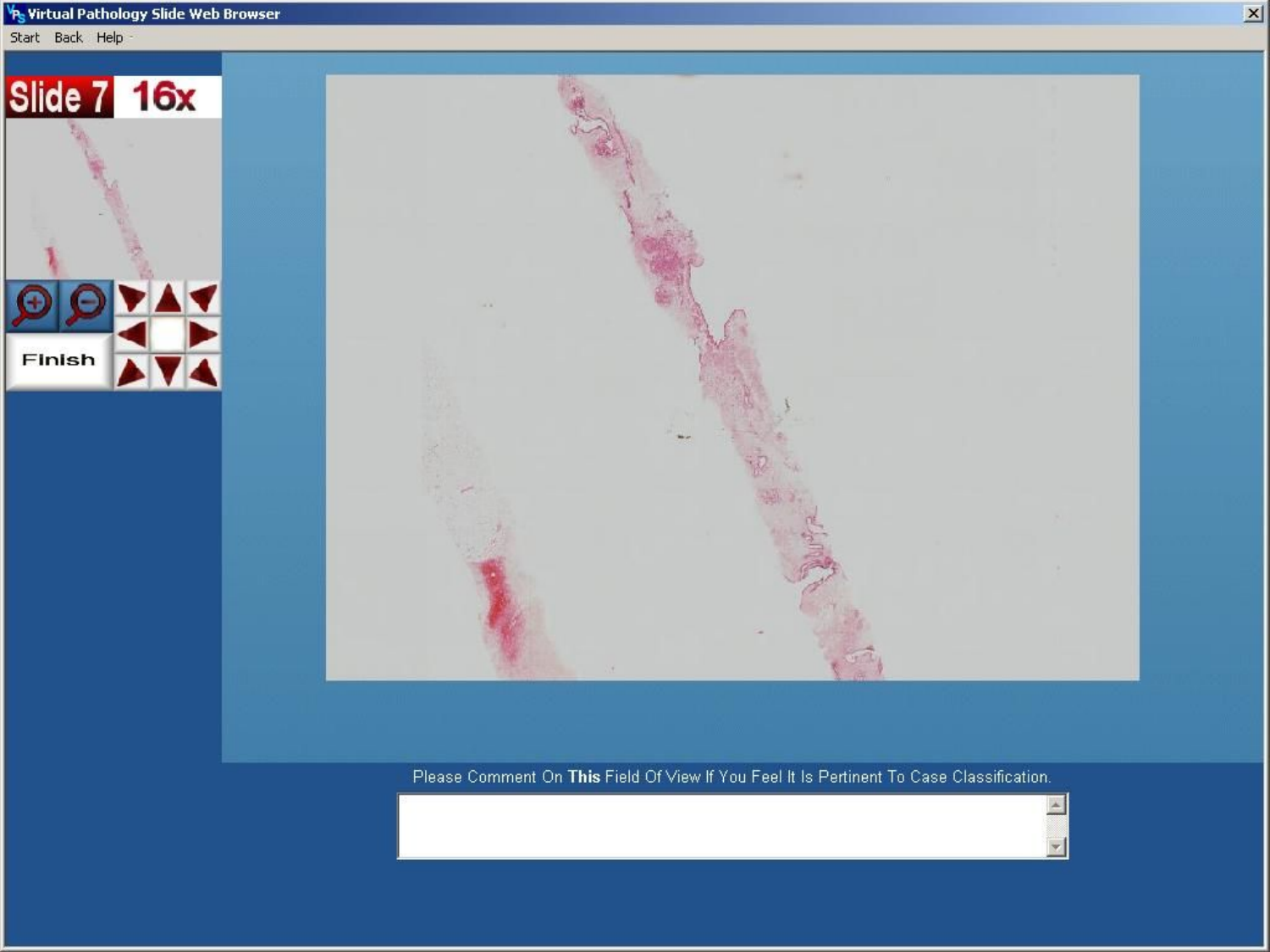

## Slide 3
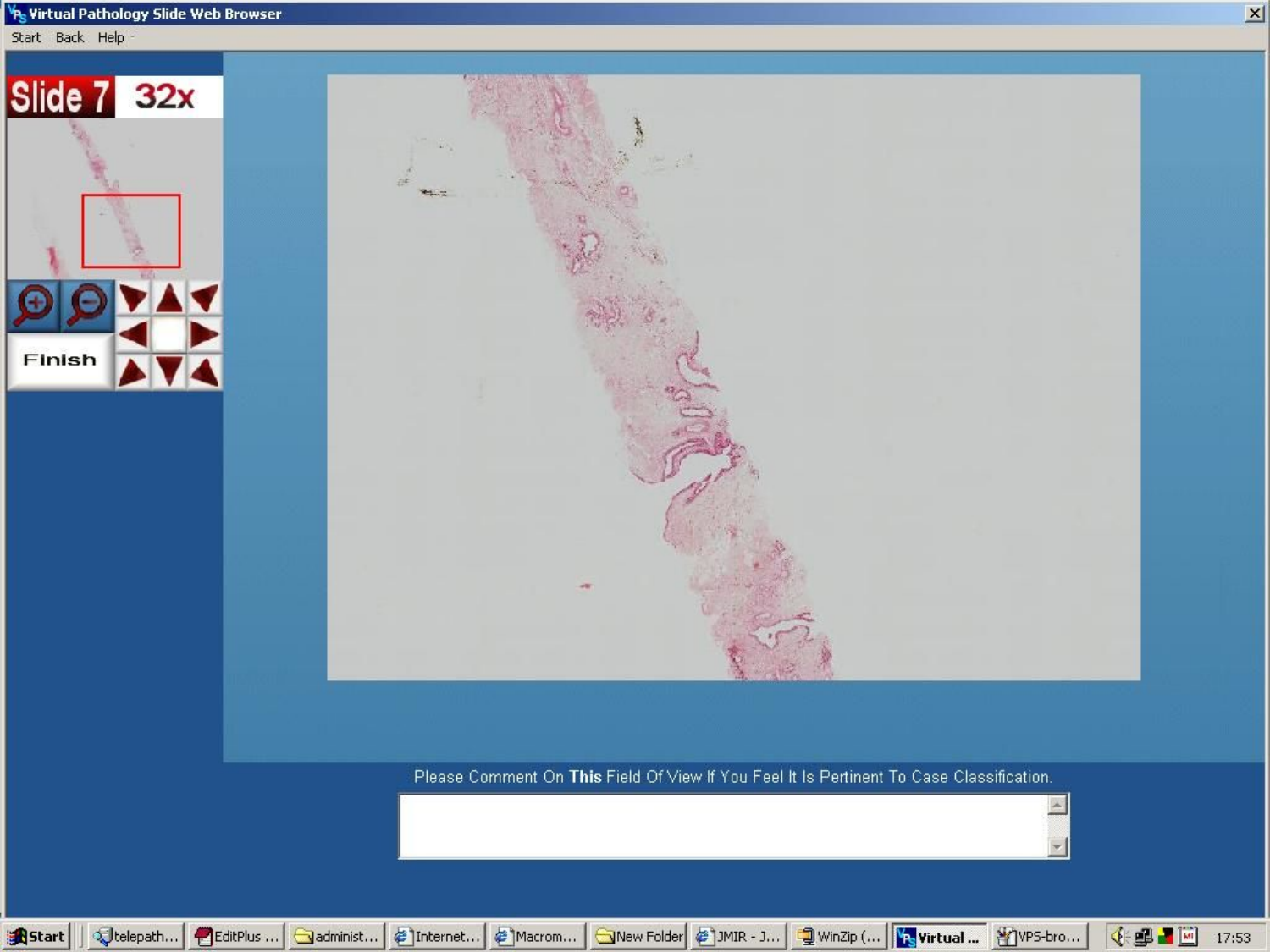

## Slide 4
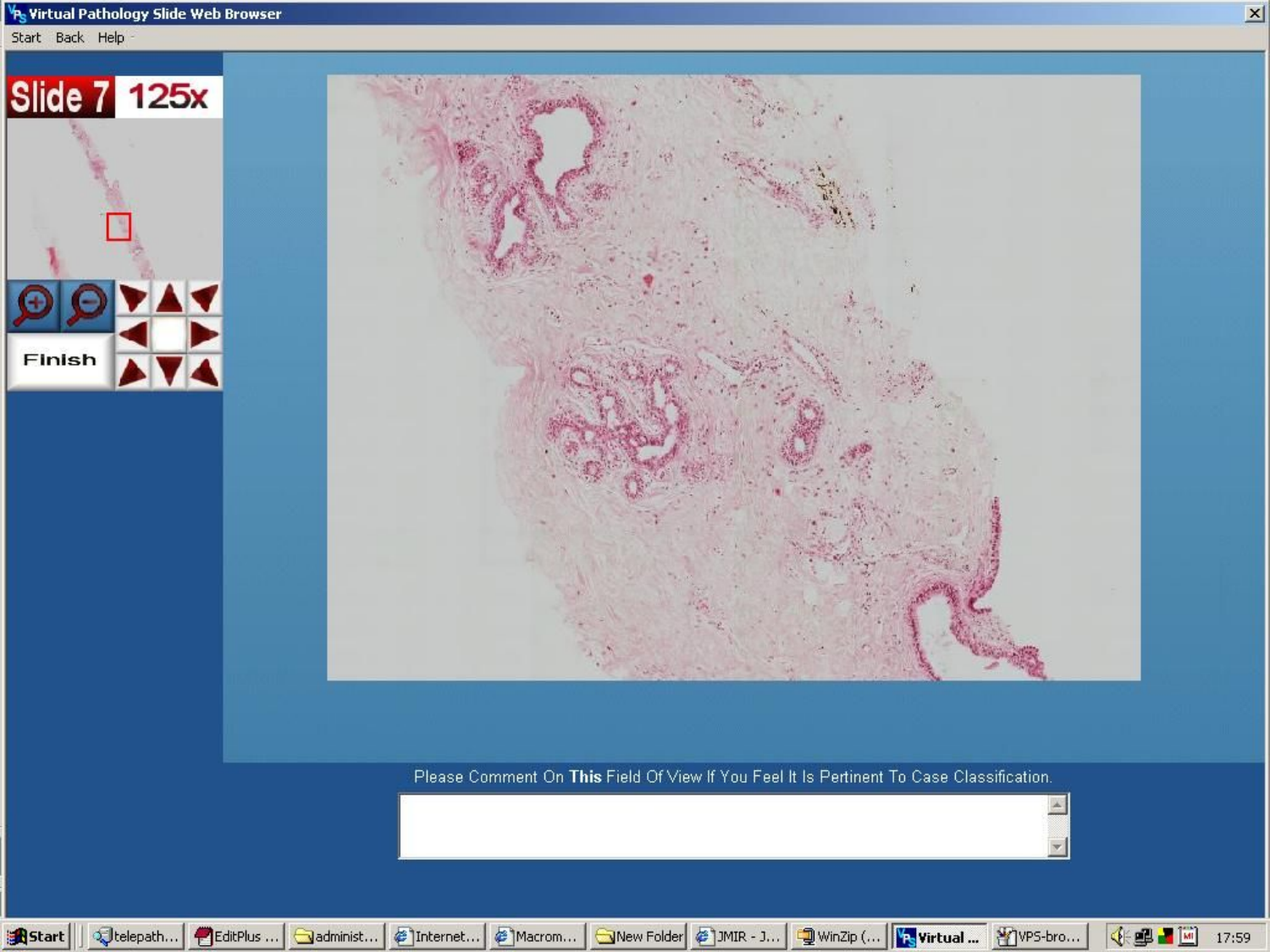

## Slide 5
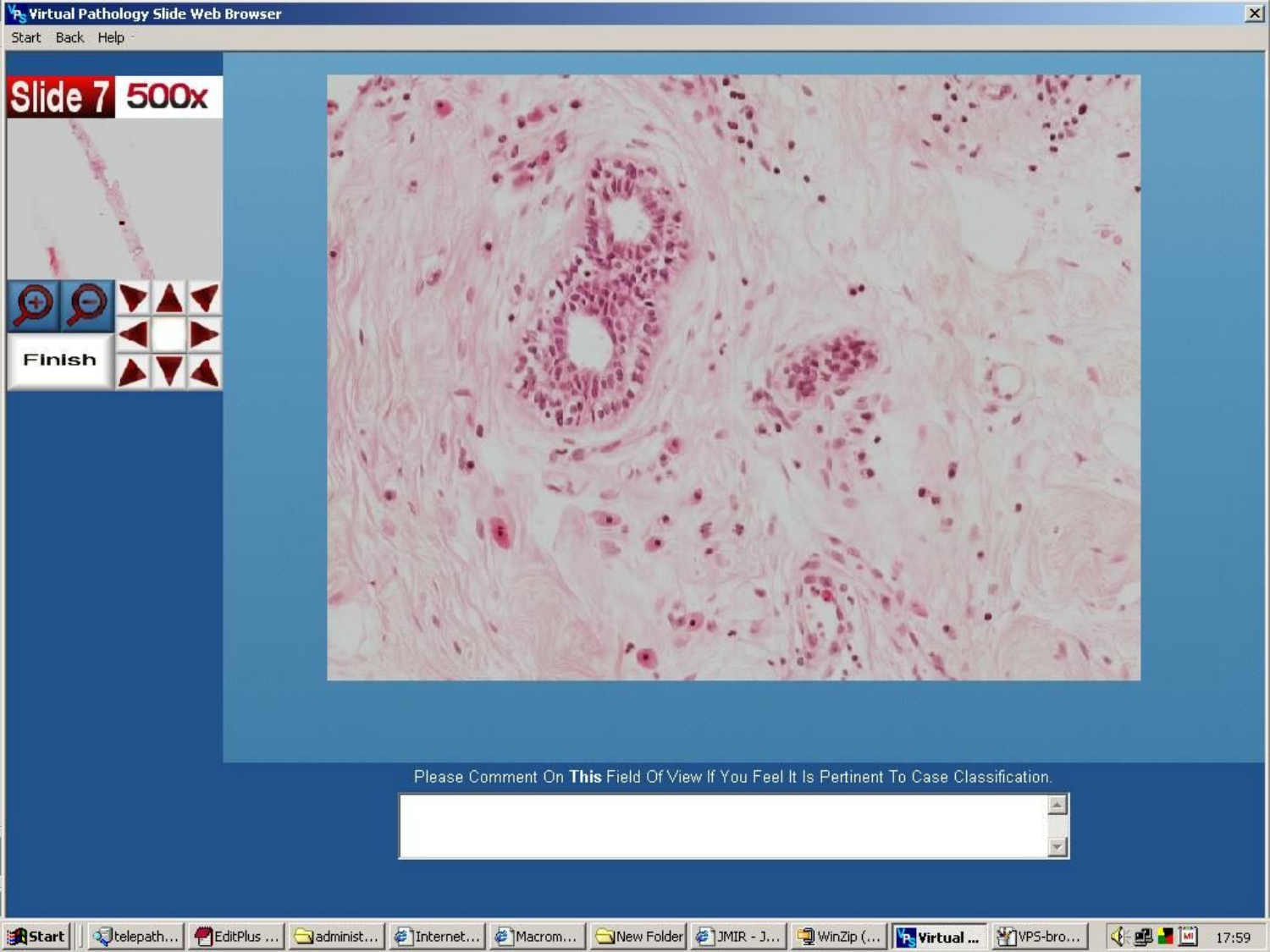

## Slide 6
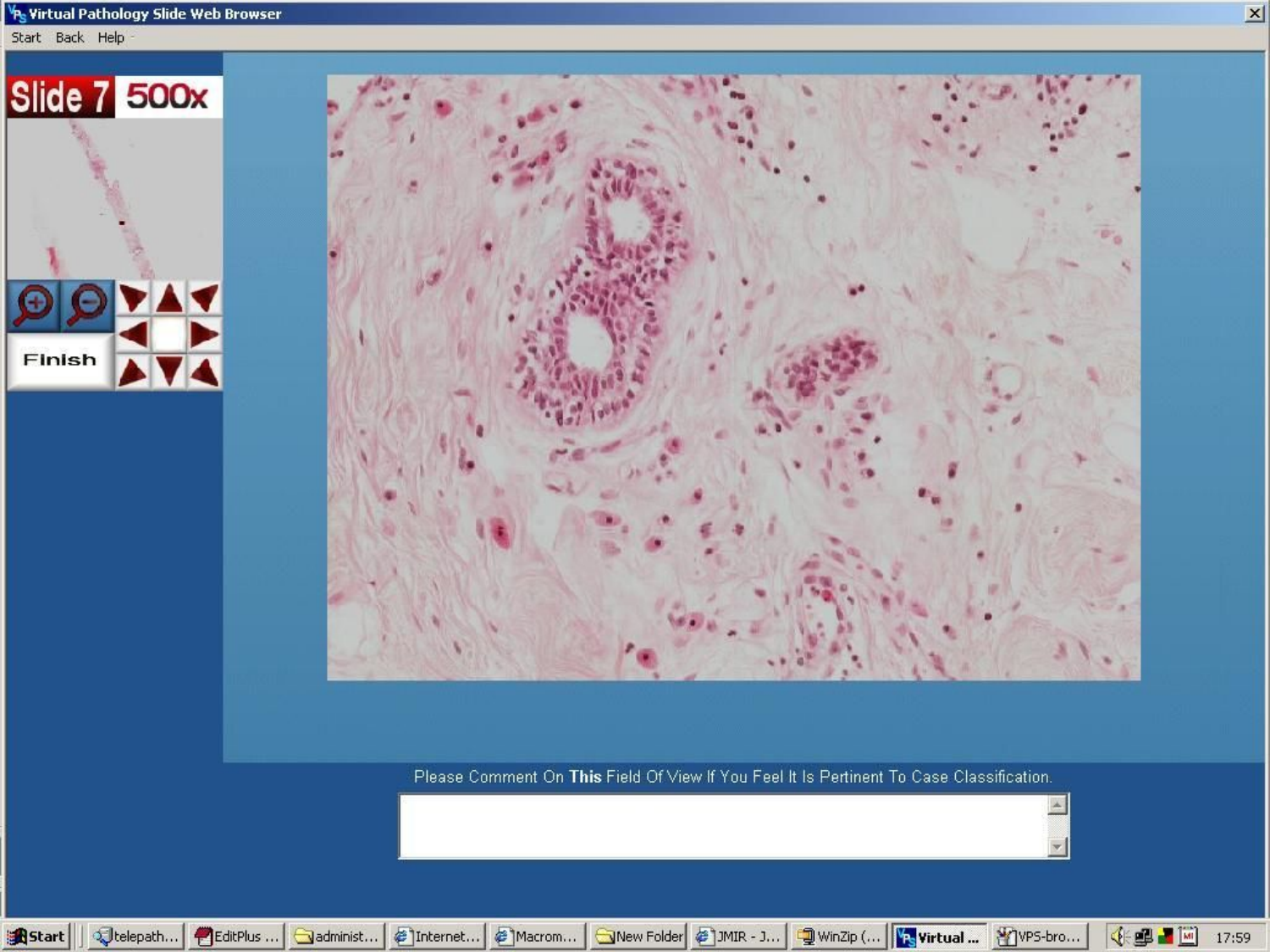

## Slide 7
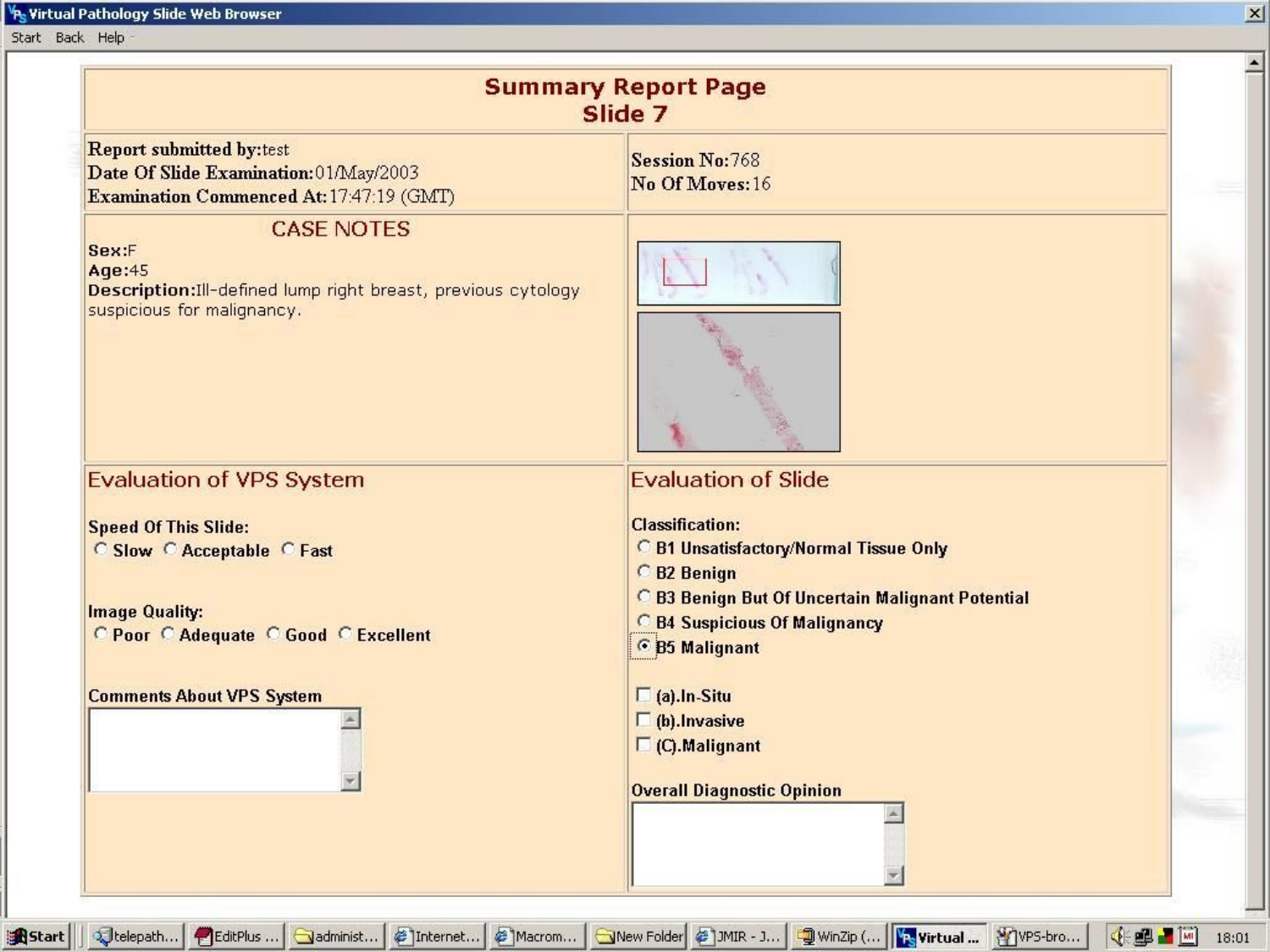

Supplement: Supplementary file 1 [file jmir_v5i2e11_app1.ppt]
