# Supplementary material for: Development and Evaluation of the Virtual Pathology Slide: A New Tool in Telepathology
Source: J Med Internet Res. 2003 Jun 13;5(2):e11. doi: 10.2196/jmir.5.2.e11 (PMC1550558; doi:10.2196/jmir.5.2.e11)
Supplement: Supplementary file 2 [file jmir_v5i2e11_app2.pdf]

Technical Document

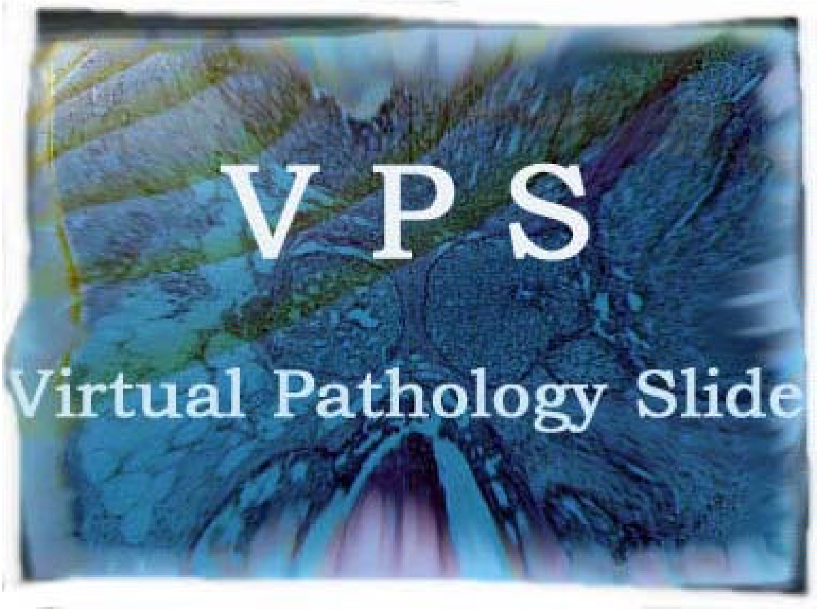

TITLE:

Technical Document.

AUTHORS:

Sean S.P. Costello[1], Daniel J. Johnston[1], Peter A. Dervan[2], Daniel G. O' Shea*[1]

RESEARCH INSTITUTIONS:

[1]Medical Informatics Group, School of Biotechnology, Dublin City University, Dublin 9, Ireland.

[2] Department of Pathology, University College Dublin and Conway Institute for Biomolecular and Biomedical Research, Dublin, Ireland.

Table of Contents

	Page.
**1.Introduction**	
1.1 Description of the VPS	3.
**2.Equipment**	
2.1 Hardware & Software Utilised	4.
**3.Construction of a Virtual Pathology Slide**	
3.1-Slide Digitisation	5.
3.2-Raster Scan - Generation Of Image-Layer 1	5.
3.3-Scaling of Image Layers 2-5	7.
3.4-File Transfer To Server	10.
**4.VPS Presentation**	
4.1-VPS Interface	12.
4.2-Client Action	14.
4.3-Server Response	14.
4.4-Client-Server Communication	15.
4.5-Zoom	18.
4.6-Zooming Out	19.
4.7-Scrolling	19.
4.8-Additional variables passed from the client to the server.	21.
**5.VPS Tracking**	
5.1-Data Recorded By Database	22.
5.2-Configuration of VPS Data Archive	22.
5.3-User Anonymity	27.
5.4-Data Retrieval	27.
**6.VPS Deployment**	
6.1-VPS Customised Browser	29.
6.2-VPS CD-ROM	29.
**Acknowledgements:**	

1. Introduction

1.1 Description of the VPS

The Virtual Pathology Slide (VPS) is an interactive microscope emulator that presents a complete digitised tissue section of size 15.53mm x 11.61mm via the Internet. VPS mimics the use of a microscope in both the stepwise increase of magnification (from 16x to 2000x) and in lateral motion in the X and Y Cartesian directions. This permits a pathologist to navigate within the selected region to a broad range of magnifications, similar to a conventional microscope.

The practice of disease classification using microscopy is a subjective skill mainly dependent on years of clinical experience by the pathologist. A novel feature of the VPS is its ability to record data sets, describing a pathologists interaction with the web-site, as they examine a pathology slide. The parameters that are recorded provide a diagnostic trace as to how and why pathologists arrive at a certain disease classification for a given case. The following document describes the methodology behind the development of the VPS.

2. Equipment

2.1 Hardware and software utilised

An imaging workstation was developed in-house to fulfil the needs of the project. An Olympus BX-40 microscope (Olympus, Melville, NY) incorporating a 40x Plan Apochromat lens with a 0.95 numerical aperture was used. The microscope was fitted with a computer controlled motorized stage (Prior Scientific Inc., Rockland MA), accurate to 0.1µm in the Cartesian X and Y planes and to 0.02µm in the Z (focus) plane, with integrated focus processor via an RS232 connection. The focus processor required a direct camera signal via a conventional video cable (S-Video signal). The video camera utilised was a JVC 3-CCD (3 chip charged couple device) with an RGB (Red Green & Blue) digital signal outputted to an Imaging Technologies IC-RGB frame grabber board. (Coreco Imaging, Incorporated, Bedford, MA). The framegrabber was housed in a personal computer with 64MB RAM and 50GB local hard drive capacity. The computer operating system utilised was Windows NT4 (service pack 6) (Microsoft Corp, Redmond, WA).

3.Construction of a Virtual Pathology Slide

3.1 Slide Digitisation

The first stage of the construction of a Virtual Pathology Slide is the digitisation of the tissue of interest at the highest required magnification (40x objective). All imaging applications were developed within Optimas 6.5, image acquisition and analysis software (Media Cybernetics, L.P., Leiden, The Netherlands). Optimas offers a programmable platform, which facilitates the creation of image analysis algorithms using ALI (Analytical Language for Images). The software also allows programmable control of RS232 interfaced devices, such as the robotic stage. An algorithm was constructed in ALI to perform a programmed raster scan of 15.53mm x 11.61mm (180mm^2) of tissue, and build subsequent layers of lesser magnification.

3.2 *Raster Scan - Generation Of Image-Layer 1*

The raster scan involves acquiring 128 x 128 images in both the X and Y Cartesian directions, one row at a time. The scanning algorithm is manually seeded with the Cartesian X, Y coordinates of the tissue midpoint. Using the midpoint coordinates, the scanning algorithm determined the boundaries of the scan.

At high power magnification, variations in tissue thickness require constant adjustment of fine focus while scanning. This is achieved using an autofocus utility integrated in the system. However, due to progressive mechanical slippage, the system had potential to lose focus over time. To compensate for this shortcoming, a custom focus determination was utilised.

Before scanning commences, the stage is manually moved to reference point within the section, representative of the topography of the entire section. An autofocus is performed at this point and a value representing the focal depth recorded. This focal value is subsequently used as a reference point during the raster scan. At the beginning of each row, the stage moves to this reference point, refocuses and recalculates a new focal depth. The offset between new and previous focal depth is a measure of the slippage that has occurred. This offset is then utilised to correct any focal depth aberration while scanning the subsequent row.

During the raster scan, for each field of view, the standard deviation of gray values is determined. If the value is above a given threshold, tissue is deemed to be present and image capture is required. A fine auto-focus is performed and the image saved. This procedure reduces processor workload and memory storage requirements for a slide, as only images that contain tissue are saved.

Two, three-digit numbers ranging from 101 to 228 are generated, describing the grid reference of each image on the Cartesian X, Y plane. These numbers are concatenated into a seven-character string separated by an arbitrary assigned delimiter (k) to form the file name for each saved image. For example

X=1 Y=2	(X, Y Cartesian coordinate of an image)
101K102.jpg	(Image file name)

Each acquired image represents 0.011 mm^2 at a resolution of 768 pixels by 574 pixels. The standardized JPEG (Joint Photographic Expert Group) image compression algorithm was utilised to save the images. A compression rate of 10% was used, resulting in image file sizes in the range of 100-150kbs.

3.3 Scaling of Image Layers 2-5

To fulfill the range of required magnifications for display, four Image Layers of lower magnification but equal resolution to Image Layer 1 are generated using a scalable imaging algorithm as described in Figure 1. The Image Layers were constructed as follows.

Image Layer 2 was constructed by resizing all the images in Image Layer 1 to 1/16 of their original size (192 pixels *by* 143 pixels).

Image Layer 3 was constructed by tiling 16 spatially related images (4 horizontal x 4 vertical) from Image Layer 2, resizing the tiled composite to 1/16 of its size and saving as one image (192 pixels *by* 143 pixels). This process was performed 1024 times to create a complete Image Layer.

Image Layer 4 was constructed in the same manner as Image Layer 3, however images from Image Layer 3 were used as the source images for all operations.
The process resulted in the production of 64 images (192 pixels *by 143* pixels)

Figure 1. -Flow diagram describing the algorithm used to construct Image Layers 2 to 5 from Image Layer 1.

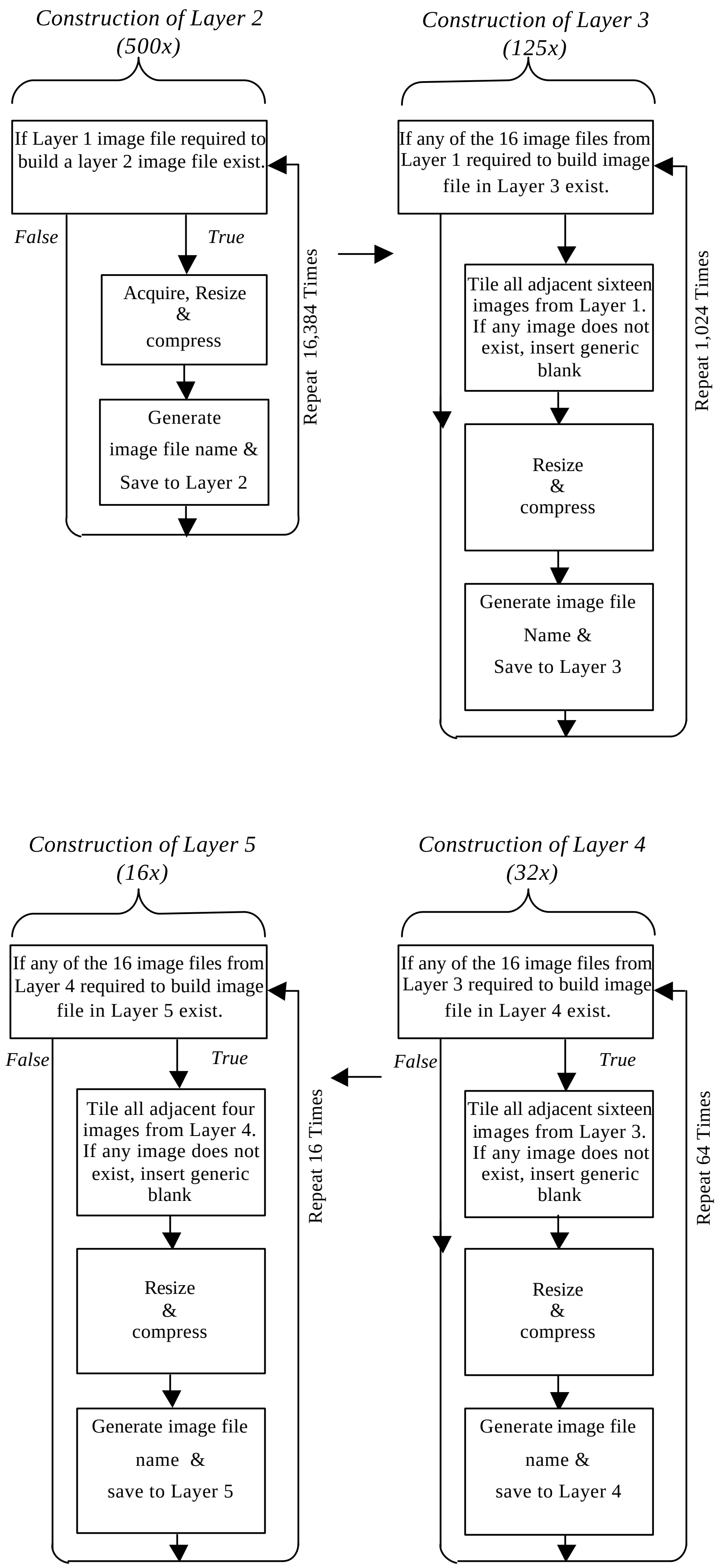

Image Layer 5 was constructed by tiling 4 spatially related images (2 horizontal x 2 vertical) from Image Layer 4, resizing them to 1/4 of their size (192 pixels *by 143* pixels) and saving as one image. This resulted in the production of 16 images with an apparent magnification of 16x.

When tiling and creating a composite image during the generation of Image Layers 3 to 5, one or more of the 16 spatially related images may not exist, as only images that contain tissue were originally saved during the raster scan. To complete the composite, a blank image generated at the beginning of the raster scan was inserted.

Image filenames for each Image Layer are generated in a similar fashion to Image Layer 1. Each Image Layer is saved to an individual folder. Images are saved as JPEG image files with a 10% compression ratio. This results in image file sizes of approximately 8kb. The total number of images generated and resultant file size for a complete digitilised VPS slide is dependent on the amount of tissue present. Typically for a needlecore biopsy 6000 images are saved. This requires approximately 600Mb of storage.

Table 1. shows the maximum possible number of images generated, image file name range and resultant magnification for each Image Layer.

Table 1. *– Composition and apparent magnification of each Image Layer within a VPS.*

IMAGE LAYER	MAGNIFICATION	*FILE NAME RANGE*	NO. OF IMAGES
1	2000x	100K100.jpeg→228k228.jpeg	16,384
2	500x	100K100.jpeg→228k228.jpeg	16,384
3	125x	100K100.jpeg→132k132.jpeg	1,024
4	32x	100K100.jpeg→108k108.jpeg	64
5	16x	100K100.jpeg→104k104.jpeg	16

3.4 File Transfer to Server

The web server utilised for the study was a Sun Microsystems Enterprise 450 (Sun Microsystems, Palo Alto, CA). It supports up to four 400-MHz/4-MB or 480-MHz/4-MB UltraSPARC-IITM processors, a 1.6-GB/sec UPA interconnect, and a 1GB/sec PCI I/O subsystem. The server can be deployed with up to 4-GB of main memory, up to 364-GB of fast hot-swap UltraSCSI internal storage, and over 6-TB of external storage capacity.

The server is connected to Ireland's academic and research network, which is known as HEAnet. HEAnet provides connections to networks in Europe by means of its 10Mbps link with the TEN-155 (Trans-European Network at 155 Megabits per second) backbone, and a 2 Mbps circuit links HEAnet to JANET, the UK education and research network. HEAnet is connected to UUnet via DANTE in New York, USA, with transit to all parts of the Internet, including a backup connection to the UK and Europe. At New York, HEAnet connects to other Internet 2 networks, including the Abilene project.

A file transfer protocol application called FTP Explorer is used to upload images to the server. This is available on the Internet at www.ftpx.com.

Image Layers are archived in an identical fashion on the server as on the local hard drive, where each layer maintains its own separate folder. The folder name has a numerical value i.e. folder “1” contains Image Layer 1.

4. VPS Presentation

4.1 VPS Interface

The VPS user interface, is a Webpage that can be displayed by any conventional browser connected to the Internet. Figure 2. illustrates the components of the VPS interface and depicts the VPS ability to zoom in on an area of interest (Figure 2(a-d)), scroll laterally (Figure 2(e)), and then zoom in to the highest magnification (Figure 2(f)). The VPS user interface is composed of sixteen images tiled seamlessly within a table of 4x4 cells. The range of apparent magnifications experienced is dependent on the user's system configuration. However, a magnification of 2000x is experienced, when using a 19 inch (48cm) monitor with a screen resolution of 1024x768. The full range of magnifications experienced at the above resolution is 2000x, 500x, 125x, 32x and 16x.

The ability to zoom in, zoom out, and scroll laterally within a VPS slide is dependent on:

(i) A client action. When the user interacts with the client, an Internet browser the user employs to view the VPS, variable input data is sent from the client to the server.

(ii) The server response. The server processes the information and dynamically creates VPS content to send to the client.

(iii) Client-Server communication, the ability of the server to receive, process and respond to variable input data from the client.

'igure 2.- Migrating through a VPS. (a) Initial view of VPS at 16x (b) Zoom to 32x on area of interest. (c) Zoom to 125x on area of interest. d) Move laterally around area of interest.(e)Zoom to 500x on area of interest.(f) Zoom to 2000x on area of interest.

i) Slide selected for viewing. (ii) Magnification of current view. (iii) Zoom and Zoom out buttons. A user may also zoom in by clicking on an nage in the VPS field of view.(iv) Lateral navigation buttons for traversing within a given magnification.(v) Auxiliary user information uttons.(iv) The VPS field of view.

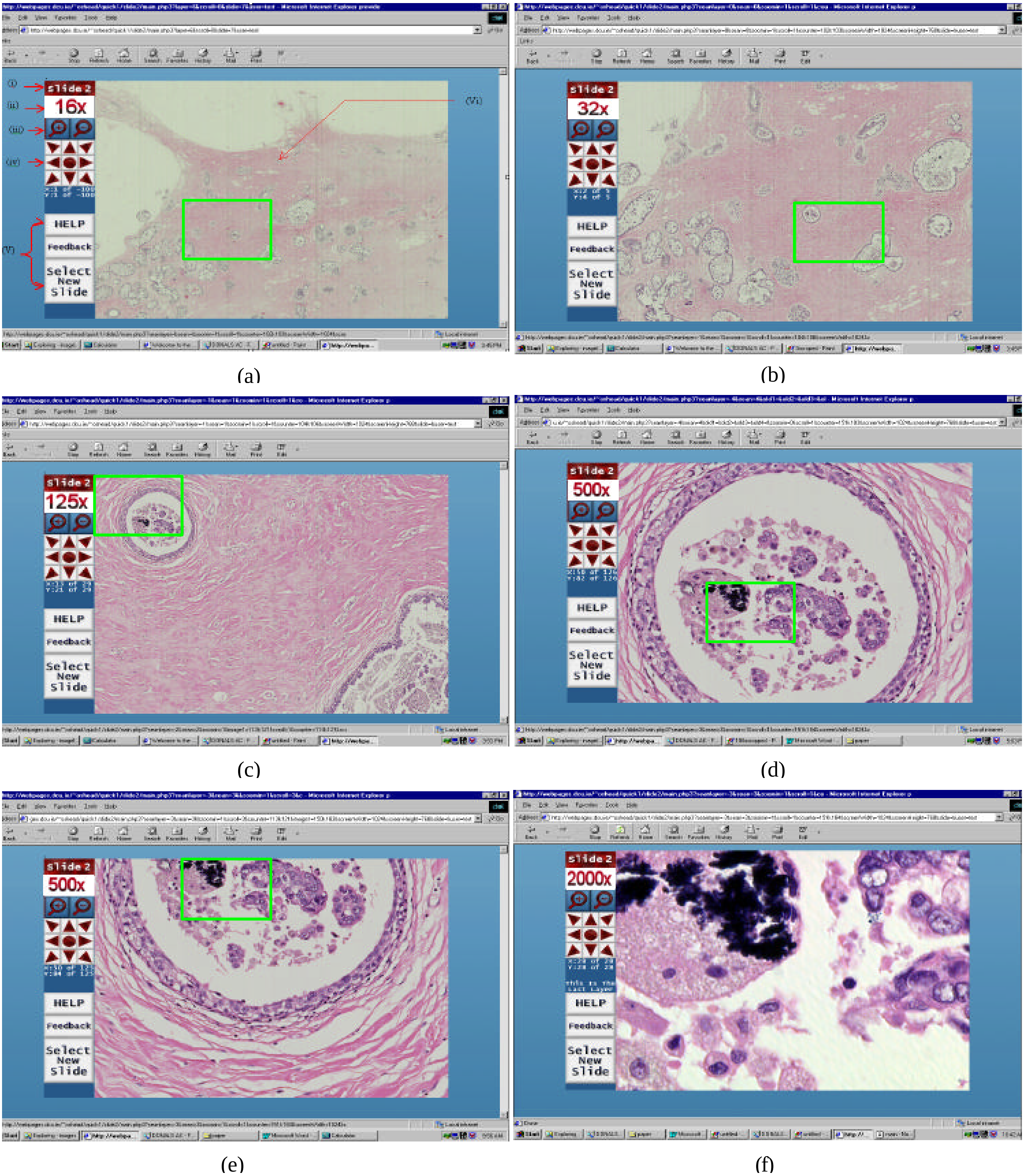

4.2 Client Action

The VPS interface is written in HTML (Hypertext Markup Language). HTML is a universal browser language for creating Webpages. It instructs a browser how to display page content, such as images and text. Acting as a client, a browser can submit to and receive data from a remote server. When a client requests a page from a server, the server sends the page to the client, with HTML instructions as to how to display the page content.

4.3 Server Response

The server is utilised as a store for information, requested by the client. Under certain conditions a server can be utilised to perform some processing at the request of the client. This feature is generally facilitated using server side scripting. Server side script is incorporated in certain HTML based pages requested by the client. To identify pages containing script, such pages are appended with a special extension (an extension other than "*.htm"). For Microsoft Active Server Pages, the extension is "*.asp", for PHP, the extension is "*.php".

PHP is an open source, server side, cross-platform, scripting language[22]. With syntax similar to the C language it has evolved from other server-side programming tools such as PERL. When the server receives a request from a client for a page with "*.php" extension, the server will open the file and parse the contents until it identifies a PHP tag within the text. The server then processes the commands within the start and finish PHP tags and replaces this content with static HTML, based on the outcome of the processing.

4.4 Client-Server Communication

For any dynamic processing to be conducted on the server, the client needs to be able to send the server variable data, which can subsequently be utilised in calculations to generate variable HTML output. The client and the server communicate using a URL. (Uniform Resource Locator). (i.e. www.telepathology.dcu.ie/index.htm)

The URL describes the location of the server on the Internet. Variables can pass between a server and a client directly within a URL, as in the following example.

www.telepathology.dcu.ie /slides/slide2/main.php3? DIRECTION=2&LAYER=3&IMAGE=101K104

These variables can be utilised within the PHP script of “main.php3”. In the above example, the variables DIRECTION, LAYER and IMAGE are passed to the page, “main.php3”. Every time the user clicks on an image or a navigational control button (Figure 2a(iv)), a hyperlink is activated calling the server page, “main.php3”. However, the value of the variables returned to the server changes, depending on the magnification the user is currently viewing, the images displayed on the VPS and the image or navigational control button the user clicked. Figure 3. illustrates the client-server communication and response for the VPS.

Figure 3. - List of server-side actions based on client side event.

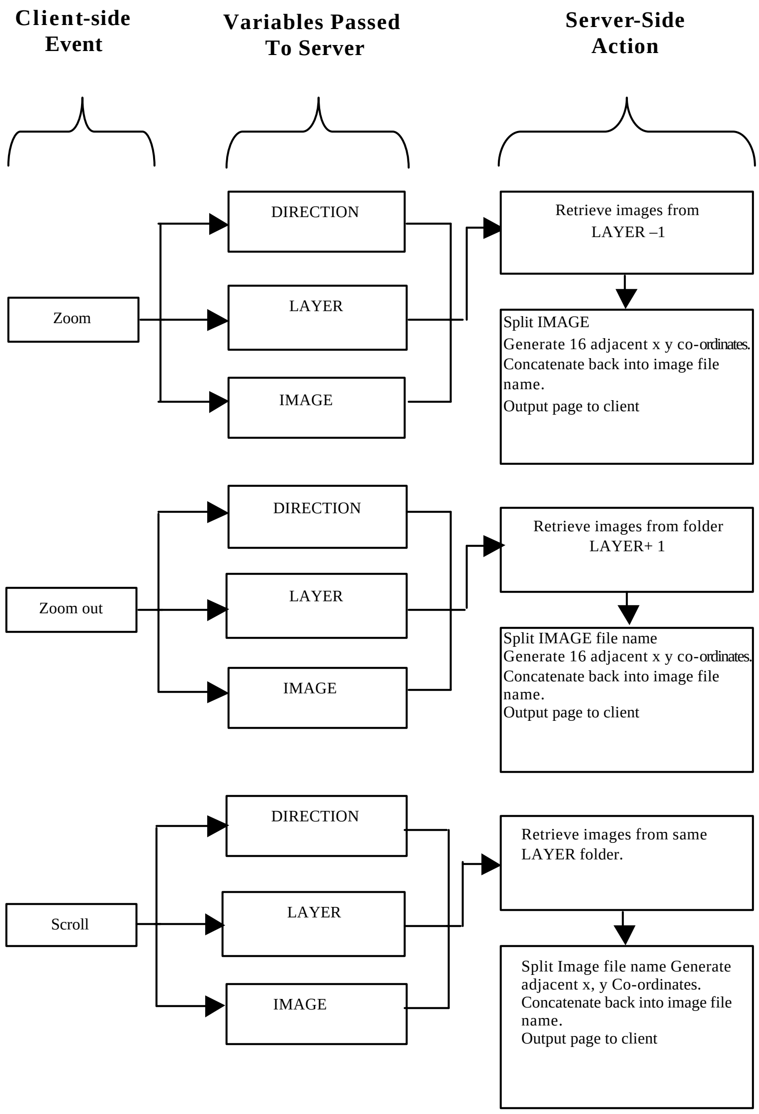

The value of the DIRECTION variable indicates the direction the user wished to migrate to (Zoom in, Zoom out, Lateral motion). On reading the value of the DIRECTION variable, the server parses the PHP script and processes only the part of the PHP script that calculates the correct set of output images for the desired move.

The value of the LAYER variable indicates the current magnification the user is viewing. It is used within the PHP script to decide which Image Layer folder to

retrieve images from, when generating a set of output images. Table 2. illustrates the possible range of values for DIRECTION and LAYER.

If the user wishes to zoom, by clicking on an image in the VPS, the value of IMAGE identifies the image the user clicked on. If the user wishes to zoom or scroll by clicking on a navigational control button (Figure 2a(iii)), IMAGE identifies the top left image of the sixteen images currently displayed on the VPS (Figure 4, "Image1"). If the user wishes to zoomout, IMAGE identifies the image that is diagonally down and to the right of the top left image for Image Layers 2 to 5 (Figure 4, "Image6"). If the user is viewing Image Layer 1 (2000x), it references the single image on display.

Figure 4.– *Structure, incorporating example of user interface for perceived magnifications, 16x, 32x, 152x, 500x.*

**Image 1**<br>x=xn y=yn<br>x=105 y=101<br>File=105K101.jpg	**Image 2**<br>x=xn+1 y= yn<br>xn=106 yn=101<br>File=106K101.jpg	**Image 3**<br>x=xn+2 y=yn<br>xn=107 yn=101<br>File=107K101.jpg	**Image 4**<br>x=xn+3 y=yn<br>xn=108 yn=101<br>File=108K101.jpg
**Image 5**<br>x=xn y=yn+1<br>xn=105 yn=102<br>File=105K102.jpg	**Image 6**<br>x=xn+1 y=yn+1<br>xn=106 yn=102<br>File=106K102.jpg	**Image 7**<br>x=xn+2 y=yn+1<br>xn=107 yn=102<br>File=107K102.jpg	**Image 8**<br>x=xn+3 y=yn+1<br>xn=108 yn=102<br>File=108K102.jpg
**Image 9**<br>x=xn y=yn+2<br>xn=105 yn=103<br>File=105K103.jpg	**Image 10**<br>xn+1 yn+2<br>xn=106 yn=103<br>File=106K103.jpg	**Image 11**<br>x=xn+2 y=yn+2<br>xn=107 yn=103<br>File=107K103.jpg	**Image 12**<br>x=xn+3 y=yn+2<br>xn=108 yn=103<br>File=108K103.jpg
**Image 13**<br>X=xn yn+3<br>xn=105 yn=104<br>File=105K104.jpg	**Image 14**<br>x=xn+1 y=yn+3<br>xn=106 yn=104<br>File=106K104.jpg	**Image 15**<br>x=xn+2 y=yn+3<br>xn=107 yn=104<br>File=107K104.jpg	**Image 16**<br>x=xn+3 y=yn+3<br>xn=108 yn=104<br>File=108K104.jpg

The client may sometimes request images that do not exist on the server, for example when viewing images around the edges of a tissue section. When this occurs, a generic blank image is inserted into the cells of the HTML table resulting in a seamless field of view.

4.5 Zoom

The first field of view the user experiences is the tissue at 16x magnification. This view is composed of 16 tiled images (4 horizontal, 4 vertical) from Image Layer 5. As the user clicks on one of the 16 images, an associated hyperlink is activated. This calls "main.php", with the variable IMAGE in the hyperlink identifying the calling image to the server.

If the server reads the variable DIRECTION=1, this results in the activation of the PHP script that constructs a field of view of higher magnification to the current field of view.

The server reads the variable LAYER (for example, LAYER=5) and decreases its value by 1 (LAYER=4). This results in the output set of images being retrieved from Image Layer 4, which contains images at 32x magnification. Using the delimiter (k) as a recognition character, PHP splits the IMAGE variable into its X and Y coordinates (e.g. IMAGE=102K104 returns X=102 Y=104). The X and Y coordinates (Xn and Yn) of the new top left image (Figure 4, "Image1") is calculated. This is based on the spatial relationship between images on different Image Layers.

The X and Y coordinates of the fifteen adjacent images are generated by manipulating the value of Xn and Yn appropriately (Figure 4), resulting in the creation of a two-dimensional reference grid. The sixteen new pairs of X and Y coordinates are concatenated with the delimiter (k) and reconstructed into a valid image file name (e.g. Xn=105 Yn=101 ∴ 105K101.jpg). A condition is included

to ensure all new image file names are valid for that particular layer. This prevents incorrect X and Y coordinates being generated and ensures that the 16 images called by the client, are within the bounds of the image set.

This method is used to zoom in until the penultimate layer (Image Layer 2) is reached. In the penultimate view (500x), if the user clicks on one of the 16 tiled images, the selected image is retrieved from Image Layer 1 (2000x) and presented to the user.

4.6 Zooming Out

When zooming out using the navigation button on the control panel (Figure 2(iii)), it is perceptually important to center the previous view within the zoomed out view. This is accomplished by assigning the previous location of Figure 4 - "Image 6", to the previous view in the zoom out view.

The server reads the variable, DIRECTION=2 (Table 2) resulting in the "zoomout" section of the PHP script being processed. The value of LAYER is increased by 1, so that image files will be retrieved from a folder containing lower magnification images.

The server reads the variable, IMAGE. As with zoom, the server splits IMAGE into its X, Y Cartesian coordinates. A condition exists to ensure that both X and Y are even numbers. If either number is not, a value of 1 is added. The Cartesian X, Y coordinates (Xn and Yn respectively) of the zoomed out top left image, is calculated using an algorithm that describes the spatial relationship between the

most central image (Figure 4, “Image 6”) on the lower-magnification Image Layer and the top left zoomed out image.

In an identical fashion to zoom in, manipulating the value of Xn and Yn generates the coordinates of the fifteen adjacent zoom-out images. Conditions within the PHP script ensure the newly created X, Y co-ordinates are valid for that Image Layer. The X and Y co-ordinates of the sixteen images are then concatenated with the delimiter and constructed into a valid image file name and attached to a dynamically created hyperlink. This results in a set of sixteen output images being sent to the client.

4.7 Scrolling

When scrolling within an Image Layer (using the navigational buttons on the control panel (Figure 2a(iv)) the following events occur. DIRECTION will have a value between 3 and 10 depending on which navigational button the user clicked (Table 2).

The server retrieves images from the same Image Layer folder (the value of LAYER remains constant). The server splits the value of IMAGE, representing the top left image file name (Figure 4, “Image 1”), into its X and Y co-ordinates. Based on the input value of the DIRECTION variable, an algorithm is used to generate the new X, Y coordinates (Xn and Yn respectively) for the top-left image of the new field of view. For example, if DIRECTION=6, the user wishes to scroll right.

(i)	X=105	Y=101	DIRECTION=6
(ii)	Xn=x+2	Yn=Y	
(iii)	Xn=107	Y=101	

In an identical fashion to Zoom, the X, Y coordinates of the fifteen adjacent images are created and concatenated back with the delimiter (k) and file format into valid file names. If the view is at 2000x (Image Layer 1), the server produces an output page containing a single image that is adjacent in the direction of scrolling, to the image in the input page.

4.8 Additional variables passed from the client to the server.

Using client-server communication, the file size of each image sent to the client, in conjunction with the time taken to download the image is recorded. This data is used to calculate the rate of data transfer per second between the server and the client. This is commonly referred to as “bandwidth” and is a descriptor of the performance of the network connection between the client and the server. When performing the calculation, data is discounted where the client has viewed an image more than once as the image may reside on the clients cache. The time spent viewing a set of images is also passed from the server to the client in order to determine the amount of time a user spent examining a field of view, as distinct to the amount of time a user spent downloading a field of view.

5. User Tracking

5.1 Data Recorded by database:

There are three specific data types recorded by the VPS.

- *System configuration data*-This consists of data submitted by a client browser to the VPS server as they interact with the VPS. This includes parameters such as client browser version, OS screen resolution, screen colour depth and IP address.
- *Client tracking data*-This data describes client "movements" as a pathologist examines a series of pathology images using the VPS. This data set is sufficiently comprehensive to replay the online examination of a VPS pathology slide by a client, in real time.
- *User submitted data*- This is data submitted to the VPS server by pathologists, using simple HTML forms.

5.2 Configuration of VPS Data Archive:

The data described above is written to an oracle database on the VPS server using PHP. The architecture of the VPS data archive is structured in a format that mirrors the sequential steps pathologists undertake when examining a VPS pathology slide.

The database is composed of seven tables. A detailed description of the database structure is described below.

- *"Users" table*- Data is submitted from a user registration HTML form. Each submission contains the following fields.

  -User e-mail　　　　　-Level of experience

-Username -Password

- *"Sessionlist" table* - Upon logging in using their username and password, a pathologist selects a slide for examination. This results in the following data specific to the slide examination being written to "*Sessionlist"*.

  -Username -Slide number

  -Server timestamp -User IP address

  -Session no -Users screen resolution

- *"Tracking" table* -Upon selecting a slide for examination a "*Tracking" table* file is created and named after the session number. This file contains a log of each move within a slide examination and records the following parameters.

  -Images viewed -Magnification Images are view at

  -VPS navigation buttons pressed -Time taken to download image

  -Time spent viewing image -IP address

  -Diagnostic Comment

- *"Sessionreport" table*. Upon completing a slide examination the pathologist submits a HTML form.

  -Username -Slide number

  -Server timestamp -User IP address

  -Session no -Users screen resolution

  - Browser details - Operating System details

  - Download speed - Image quality

  -Comment on image quality - Classification of slide

- Sub classification of slide
- Diagnostic Comment

- *"Feed" table* Contains data submitted from an online feedback form. This contains user perceptual data of the VPS and is based on a questionnaire.
  - Username
  - IP Address
  - Server timestamp
  - User Comments

Schematic Indicating Origin Of Data Stored in VPS Database

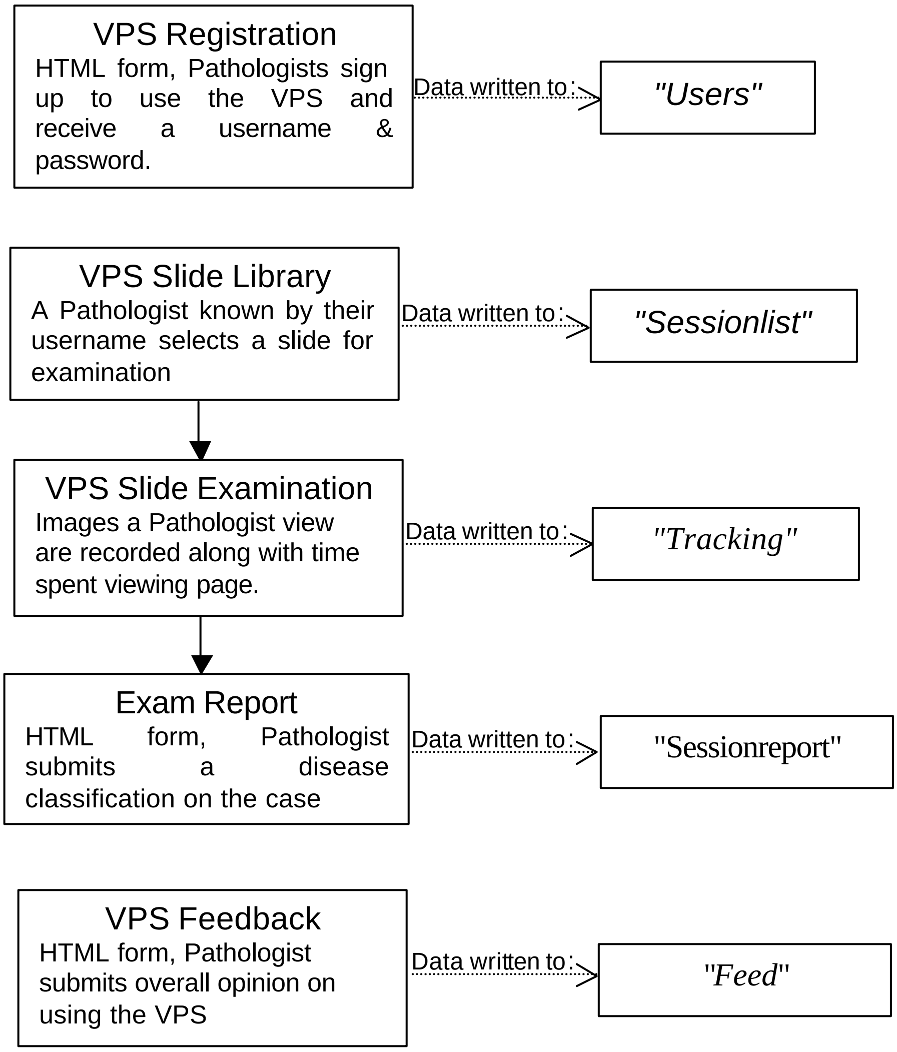

Table Content for Oracle Database

Table Name	Column Name	Description
**User**	UserId	Data submitted from user registration HTML form
	UserName	
	Password	
	Date	
	E-mail	
	Experience	
**Session List**	UserId	Data submitted when a logged in user selects a pathology slide for examination.
	Time	
	Date	
	SlideNo.	
	SessNo.	
	TrackNo.	
	MachNo.	
**Machine Spec**	MachNo.	Data submitted when a logged in user selects a pathology slide for examination.
	ScreenH	
	ScreenW	
	BrowserOs	
	Color size	
**Table Name**	**Column Name**	**Description**
**Tracking**	TrackNo.	Data submitted while a logged in user examines a pathology slide.
	Magnif	
	SlideX	
	SlideY	
	Button	
	Download	

	TimeTaken	
	Time	
	TimeView	
**Session Report**	SessNo.	Data submitted from user via a HTML form upon completing examination of a pathology slide.
	Bandwidth	
	Time	
	Dspeed	
	ImageQ	
	Comment	
	Class	
	SubClass	
	Diagnosis	
**FeedBack**	UserId	Data submitted from a user via a HTML form upon examination of a set number of pathology slides.
	Date	
	Competence	
	Frequency	
	ImageQ	
	PrepQual	
	DownlSp	
	EaseUse	
	Confidence	
	WhyNotCom	
	Improve	
	Comment	

5.3 User Anonymity:

Upon registration, every user is assigned a unique user id number. When examining a VPS slide it is this number that is associated with the data that is recorded in the VPS database. This preserves their anonymity and ensures any data submited to the server can be stored in the strictest confidence.

5.4 Data Retrieval:

In order to monitor in-house, users interaction with the VPS and perform statistical analysis of the data, a website powered by a PHP engine to successfully interrogate data for statistical analysis was developed. The php engine dynamically writes SQL queries which retrieves requested information from the database.

6. VPS Deployment

6.1 VPS Customised Browser

The VPS customised browser was developed to control access for users during VPS dedicated studies, optimise the integrity of recorded data and to provide a uniform experience for users who would otherwise experience subtle differences due to the plethora of web browser versions currently in existence.

Constructed using Visual C++ application wizard, the VPS Customised Browser is a Microsoft Foundation Class (MFC) application that utilises Internet Explorer file libraries in order to behave as a customised browser. The VPS Customised Browser opens up a prescribed webpage on the VPS server. The VPS browser is optimised for PC users with Internet Explorer 5.

3.2 VPS CD-ROM

The availability of high-speed broadband Internet connectivity is currently still unavailable to many pathologists. This is currently a major limiting factor for acceptability of the web driven telepathology due to the time taken to download large number of images over the Internet. In order to improve users experience, the VPS interface has been adapted to download images locally from a VPS-CD, if one is present in the CD-ROM drive. Otherwise images are downloaded from the VPS server.

Acknowledgements:

The authors would like to thank the Irish Health Research Board (HRB), for their partial funding of this work. The authors would also like to thank the pathologists who took time to validate this technology, and provided such useful and constructive feedback.
